# Supplementary material for: Prognostic Significance of Tumor Regression Rate during Concurrent Chemoradiotherapy in Locally Advanced Cervix Cancer: Analysis by Radiation Phase and Histologic Type
Source: J Clin Med. 2020 Oct 28;9(11):3471. doi: 10.3390/jcm9113471 (PMC7692078; doi:10.3390/jcm9113471)
Supplement: Supplementary file 1 [file jcm-09-03471-s001.pdf]

Journal of
Clinical Medicine

MDPI

Supplementary material

Table S1. Univariate analysis of prognostic factors for PFS/OS.

Characteristics	PFS		OS	
	**Hazard Ratio (95%, CI)**	***p*-Value**	**Hazard Ratio (95%, CI)**	***p*-Value**
Cell type				
SCC	11		1	
AC/ASC	2.74 (1.71-4.40)	< 0.001	2.41 (1.53-3.78)	< 0.001
Pretreatment Hb				
< 11 (g/dL)	1		1	
≥ 11 (g/dL)	0.95 (0.63-1.44)	0.826	0.87 (0.59-1.27)	0.481
Stage				
II	1		1	
III	1.72 (1.13-2.62)	0.010	1.92 (1.30-2.85)	0.001
IV	1.68 (0.93-3.04)	0.053	1.89 (1.09-3.25)	0.022
LN metastasis				
Negative	1		1	
Positive	1.45 (0.96-2.19)	0.075	1.34 (0.90-1.98)	0.144
Tumor size				
≤ 4.0 cm	1		1	
> 4.0 cm	1.41 (0.87-2.28)	0.153	1.37 (0.88-2.13)	0.163
$EBRT_{regression}$				
> 26%	1		1	
≤ 26%	2.29 (1.50-3.51)	<0.001	2.52 (1.71-3.71)	<0.001
$EBRT_{proportion}$				
> 40%	1			
≤ 40%	1.54 (1.36-1.80)	0.002	2.16 (1.50-3.11)	<0.001

PFS, progression-free survival; OS, overall survival, SCC, squamous cell carcinoma; AC, adenocarcinoma; ASC, adenosquamous cell carcinoma; Hb, hemoglobin; LN, lymph node; EBRT, external beam radiotherapy; $EBRT_{regression}$, post-EBRT tumor size regression rate; $EBRT_{proportion}$, proportion of EBRT to overall size regression.

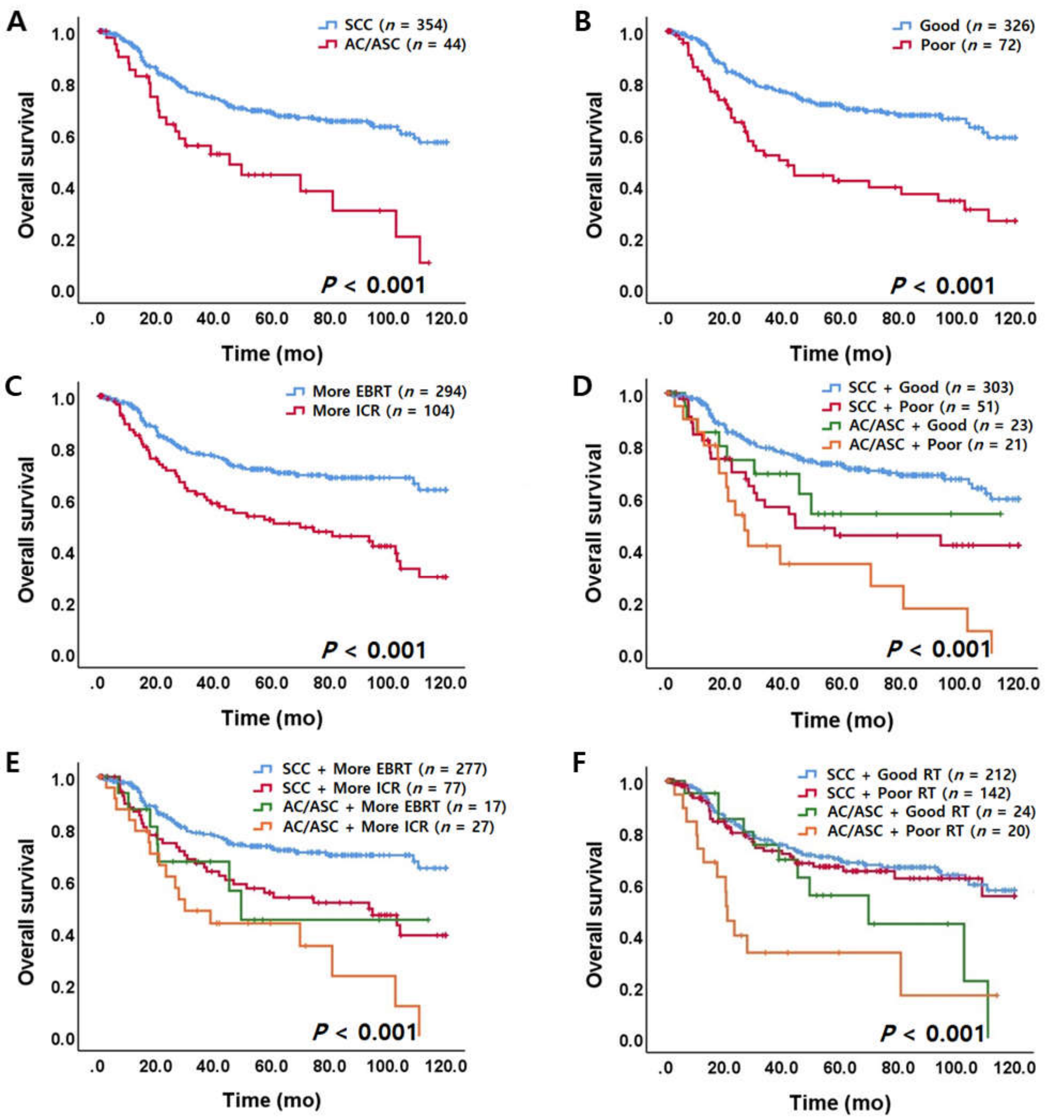

Figure S1. Overall survival (OS) according to histologic subtype and responsiveness to RT. (A) OS according to histologic subtype. (B) OS according to EBRTregression. (C) OS according to EBRTproportion. (D) OS according to histologic subtype and EBRTregression. (E) OS according to histologic subtype and EBRTproportion. (F) OS according to histologic subtype and RTregression. RT, radiation therapy; SCC, squamous cell carcinoma; AC, adenocarcinoma; ASC, adenosquamous carcinoma; EBRT, external beam radiotherapy; ICR, intracavitary brachytherapy; EBRTregression, post-EBRT tumor size regression rate; EBRTproportion, proportion of EBRT to overall size regression; RT regression, overall regression rate after completion of RT; Good, good EBRT responder (EBRTregression > 26%); Poor, poor EBRT responder (EBRTregression ≤ 26%); More EBRT, more EBRT responders (EBRTproportion > 40%); More ICR, more ICR responders (EBRTproportion ≤ 40%); Good RT, good RT responders (RTregression > 92%); Poor RT, poor RT responders (RTregression ≤ 92%).

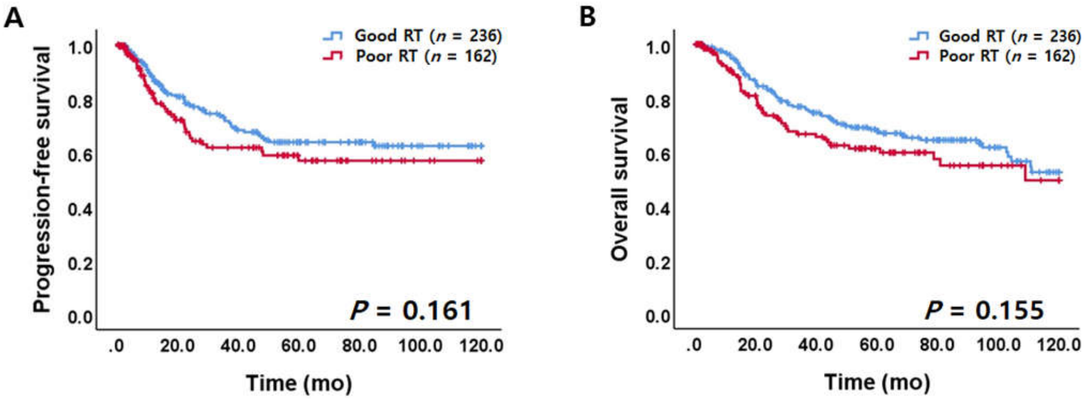

Figure S2. Survival outcomes according to $RT_{regression}$. (**A**) PFS according to $RT_{regression}$. (**B**) OS according to $RT_{regression}$. PFS, progression-free survival; OS, overall survival; RT, radiation therapy; $RT_{regression}$, overall regression rate after completion of RT; Good RT, good RT responders ($RT_{regression} > 92\%$); Poor RT, poor RT responders ($RT_{regression} \leq 92\%$).

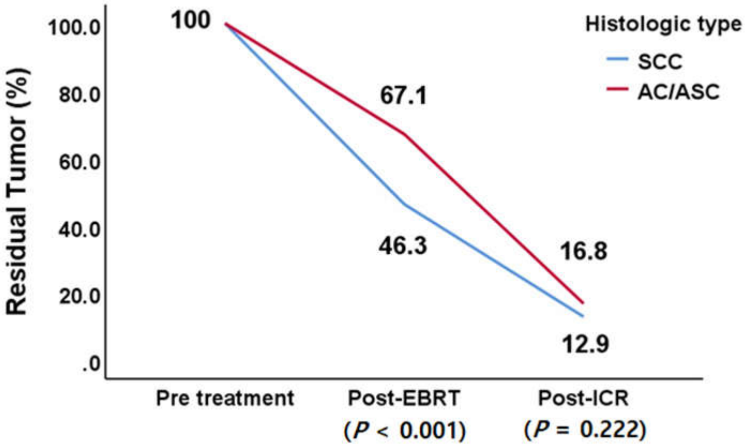

Figure S3. Tumor regression pattern according to histologic subtype and RT phase. SCC, squamous cell carcinoma; AC, adenocarcinoma; ASC, adenosquamous carcinoma, EBRT, external beam radiotherapy; ICR, intracavitary brachytherapy.
